# Breaking the Emergency Room Cycle: The Impact of Telemedicine on Emergency Department Utilization

**DOI:** 10.7759/cureus.55457

**Published:** 2024-03-03

**Authors:** Kareema Alshurtan, Heba Almomtin, Khaled F Alqhtani, Abdullah Alqahtani, Amirah Aledaili, Amani Alharbi, Mawaddah Alhejaili, Shatha H Alreheili, Shadan Aljassar

**Affiliations:** 1 Internal Medicine, University of Hail College of Medicine, Hail, SAU; 2 Medicine, University of Hail College of Medicine, Hail, SAU

**Keywords:** telemedicne, effectiveness, awareness, ehealth impact, emergency department

## Abstract

Background

Telemedicine has gained prominence in healthcare, and understanding its impact on diverting non-urgent cases from the emergency room (ER) has become crucial. This study delves into the dynamic relationship between telemedicine utilization and ER visits, seeking to understand the transformative impact of telehealth on breaking the traditional ER cycle.

Method

To explore the awareness and utilization of telemedicine services in the broader population of Saudi Arabia, we adopted a cross-sectional design utilizing the convenience sampling method. Data collection was facilitated through a self-administered online survey comprising four sections: demographic variables, ER visits, awareness of non-urgent cases, and suggestions. The collected data were entered into IBM SPSS Statistics for Windows, Version 21 (Released 2012; IBM Corp., Armonk, New York, United States) for descriptive analysis.

Results

Among the 1140 participants, the majority were females (56.8%), with 43.2% being males. Regarding age distribution, the highest percentage fell within the 18-25 age group (46.8%). Nationality-wise, a substantial proportion identified as Saudi (95.2%). Educational backgrounds varied, with 60.7% holding a bachelor's degree. Notably, 62.6% of the visits were classified as urgent. A significant portion (66.8%) demonstrated awareness of alternative options for non-urgent care, such as outpatient clinics and telemedicine services. Specifically regarding telemedicine, 82.8% of respondents believed that video consultations and prescription refills could effectively address non-urgent conditions. Furthermore, 89.6% of participants agreed that educating patients on self-care and home remedies could help manage symptoms and deter unnecessary ER visits.

Conclusions

The positive reception of telemedicine, as evidenced by high satisfaction rates among participants, further reinforces its role in reshaping the landscape of healthcare delivery.

## Introduction

Emergency departments/emergency rooms (EDs/ERs) began with the advent of hospital-based medicine following World War II. They serve as crucial hubs for acute ambulatory and inpatient care in contemporary healthcare [1]. As, emergency medical diseases (EMDs) have played a significant role in causing 50.7% of mortalities, accounting for 41.5% of the overall burden of diseases. Notably, the burden of EMDs in low-income countries is 4.4 times higher than in high-income countries [2]. The overcrowding issue in ER is a significant concern in healthcare, drawing heightened attention from the public and regulatory bodies globally [3], particularly in Saudi Arabia. Nevertheless, another significant factor contributing to the burden on ERs is the surge in non-urgent and less-urgent patients seeking care in the ED setting. This challenge often stems from a lack of patient education or accessibility to alternative services [4]. Patients classified as less-urgent or non-urgent are widely dispersed across various districts within a city. Consequently, directing these patients to the ER exacerbates overcrowding, potentially impeding or diminishing the effectiveness of healthcare interventions for individuals with severe illnesses requiring resuscitation, emergent, or urgent medical attention [5]. Furthermore, ER overcrowding can diminish patient satisfaction and heighten their risk vulnerability [5]. Various strategies have been explored to address these pressing challenges [3]. Meanwhile, the rapid advancement of telemedicine in delivering healthcare services has significantly reduced overcrowding [1,6]. In recent years, the impact of telemedicine on ER utilization has become a subject of considerable interest and scrutiny, particularly in the context of the evolving landscape of healthcare delivery [7]. Meanwhile, telemedicine, defined as the remote delivery of healthcare services using technology, has gained prominence as a viable alternative to traditional in-person care, offering the potential to enhance accessibility, efficiency, and cost-effectiveness [6]. In Saudi Arabia, the Ministry of Health has implemented innovative telemedicine technology to cater to patients through mobile applications, such as the Sehhaty app and the 937 medical call center [8]. However, it is essential to enhance public awareness regarding the significance of telemedicine in Saudi Arabia.

Several studies have demonstrated a positive correlation between telemedicine adoption and decreased non-emergent ER visits [9]. The convenience of virtual consultations and the ability to access healthcare from the comfort of one's home has proven attractive to individuals seeking medical advice for minor ailments or routine follow-ups [10]. Telemedicine has been particularly effective in managing chronic conditions, allowing for continuous monitoring and timely interventions without needing in-person ER visits [11]. However, challenges persist in the widespread adoption of telemedicine, including issues related to technology access, digital literacy, and concerns about the quality of virtual care [12]. Disparities in technology access and digital literacy may contribute to unequal utilization patterns, with certain demographic groups facing barriers in adopting telehealth services. Additionally, telemedicine is well-suited for many non-urgent medical issues; there are instances where in-person evaluations in the ER remain essential, especially during COVID-19 [13]. Striking a balance between the convenience of telemedicine and the necessity of in-person care is crucial for optimizing healthcare delivery and ensuring patient safety. Moreover, the impact of telemedicine on ER utilization extends beyond individual practices to broader systemic implications. Health policymakers and administrators must consider the integration of telehealth into existing healthcare frameworks, addressing regulatory, reimbursement, and infrastructure challenges [14]. The success of telemedicine in reducing non-emergent ER visits depends on a multifaceted approach that involves not only individual adoption but also systemic support and integration [15].

The utilization of ER is often marked by an influx of non-urgent cases, contributing to overcrowding and straining healthcare resources. Introducing telemedicine as an alternative approach holds the potential to alleviate this burden by offering a more accessible and efficient means of healthcare delivery. The rationale for such a shift is rooted in the need to create awareness among the population of Saudi Arabia about the advantages of telemedicine. By fostering understanding and acceptance of remote healthcare solutions, individuals can be empowered to seek timely medical advice for non-emergent concerns through virtual consultations, reducing unnecessary visits to the ER. This awareness-driven approach aligns with the broader goal of optimizing healthcare resources, enhancing patient experiences, and contributing to a more sustainable and responsive healthcare system in the context of Saudi Arabia. Thus, the present aimed to assess the awareness of the Saudi public regarding the impact of telemedicine on ED utilization.

## Materials and methods

Study design

To investigate the awareness and utilization of telemedicine services within the general population of Saudi Arabia, a cross-sectional design was followed. This research methodology enables the gathering of an extensive dataset, facilitating a comprehensive exploration of the research objectives [16].

Study population and sampling

For the sample selection in this study, the convenience sampling method was employed, a pragmatic technique enabling the selection of participants based on their availability or the researcher's convenience [17]. The study included hospitals across Saudi Arabia. An electronic questionnaire was distributed via social media platforms to residents of Saudi Arabia with experience working in Ministry of Health hospitals and private hospitals throughout the kingdom. Consequently, the sample was chosen by adhering to specific inclusion criteria, encompassing individuals from Saudi and non-Saudi populations, male and female, aged 18 and above, and expressing an interest in participating in the study. The sample size was determined using the following formula:

Sample Size = (Z^2^×P×(1-P)/e^2^)​/1+(Z^2^×P×(1-P)/e^2^N)

In the context of statistical considerations, where N represents the total population, P denotes the standard deviation, e signifies the margin of error (5%), and z represents the z-score (1.96 at a 95% confidence level), it has been determined that the minimum required sample size is 1140.

Data collection

The study employs a self-administered online survey consisting of four segments for data collection purposes. The initial section focuses on sociodemographic information, encompassing gender, age, nationality, education level, residency, income, reasons for ER visits, classification of visits as urgent or non-urgent, and referrals. The subsequent section was dedicated to inquiries about participants' ER visit experiences. The third section comprises structured questions to evaluate participants' awareness of non-urgent cases. Finally, the fourth section encompasses queries addressing participants' perspectives on the utilization of telemedicine.

Data analysis

The collected data were entered into IBM SPSS Statistics for Windows, Version 21 (Released 2012; IBM Corp., Armonk, New York, United States) for descriptive analysis. Quantitative data was represented as numerical values and percentages across variables.

Ethical consideration

Ethical considerations in the study were paramount and approved by the Research Ethics Committee at the University of Hail on June 5, 2023 (with reference number: H-2023-309). Protecting participants' rights, privacy, and confidentiality was of utmost importance, given the sensitive nature of healthcare data. Informed consent, transparency, and voluntary participation were integral to upholding ethical standards. Researchers were also vigilant in safeguarding the integrity of the data collected, utilizing secure storage and transmission methods.

## Results

There were 1140 participants, and most of them were females (56.8%), while 43.2% were males. Age distribution reveals that the highest percentage falls within the 18-25 age group (46.8%), followed by 25-35 (21%), 35-45 (18.2%), and more than 45 (14%). Nationality-wise, a significant proportion were Saudi (95.2%), with a small percentage being non-Saudi (3.5%). Educational background varied, with 60.7% having a bachelor's degree, 24.6% completing high school, 8.2% holding a diploma, and 6.4% having higher education. Geographically, individuals from the northern region (21.1%) and central region (20.2%) constituted the highest proportions among residency locations. The monthly family income distribution indicates that 32.9% had an income greater than 15000 Saudi riyals. Reasons for ER visits include abdominal pain (22.9%), common cold (18.1%), and chest pain (10.4%). Most visits were classified as urgent (62.6%), and a substantial number sought medical care without prior consultation (75.2%). Referrals from primary health care were reported by 23.4% of the participants (Table 1).

**Table 1:** Demographic characteristics of participants

Characteristics	Frequency	Percentage
Gender		
Male	493	43.2
Female	647	56.8
Age (years)		
18-25	534	46.8
25-35	239	21
35-45	207	18.2
More than 45	160	14
Nationality		
Saudi	1085	95.2
Non-Saudi	40	3.5
Education level		
High school	281	24.6
Diploma	94	8.2
Bachelor’s degree	692	60.7
Higher education (Master/PhD)	73	6.4
Residency		
Central region	230	20.2
Western region	202	17.7
Eastern region	233	20.4
Northern region	240	21.1
Southern region	235	20.6
Monthly family income (Saudi riyal)		
Less than 5000	176	15.4
5000-10000	287	25.2
10000-15000	302	26.5
More than 15000	375	32.9
Reasons for the ER visit		
Common cold	206	18.1
Chest pain	118	10.4
Abdominal pain	261	22.9
Headache	105	9.2
Lower back pain	80	7.0
Rearrange missing appointment	24	2.1
Refilling medication	67	5.9
Peripheral numbness	1	.1
Trauma	122	10.7
Neurologic symptoms	1	.1
Others	153	13.4
Urgent or non-urgent visit		
Urgent	714	62.6
Non-urgent	426	37.4
Seek medical care before visiting the ER		
Yes	283	24.8
No	857	75.2
Referred from primary health care		
Yes	267	23.4
No	873	76.6

The first section outlines participants' familiarity with non-urgent cases in the ER, with 52.5% indicating knowledge of such cases. Most respondents (86.6%) believe non-urgent ER visits contribute to issues like overcost, overcrowding, and longer wait times. Additionally, 82.6% know that visiting the ER for non-urgent conditions increases the risk of exposure to infectious diseases and medical errors. Furthermore, 82.2% recognize the benefits of seeking medical care for non-urgent conditions outside the ER, such as shorter wait times and access to specialized physicians (Table 2). A significant portion (66.8%) were aware of alternative options for non-urgent care, including outpatient clinics and telemedicine services. Regarding telemedicine, 82.8% of respondents believe that video consultations and prescription refills can effectively address non-urgent conditions. The subsequent section provides frequencies and percentages for specific non-urgent conditions, highlighting that the common cold is the most prevalent concern at 77.9%. Finally, Table 2 addresses misconceptions about the ER, with a notable proportion believing that all medical conditions are emergencies (44.4%) and that the ER is the wrong place for medication refills and non-urgent cases (22.7%). There are also misconceptions about the ER being the only 24/7 healthcare facility (20.4%) and having longer wait times than other options (12.5%).

**Table 2:** Awareness of non-urgent cases

Questions	Frequency (%)	
	Yes	No
Do you know the non-urgent cases in the emergency room?	598 (52.5)	519 (45.5)
Do you think that non-urgent emergency room visits contribute to overcost, overcrowding, and longer wait times?	989 (86.6)	151 (13.2)
Are you aware that if you visit the emergency room for a non-urgent condition, your risk of being exposed to infectious diseases and medical errors will be higher?	942 (82.6)	198 (17.4)
Are you aware that shorter time waiting and getting access to specialized physicians outside the emergency room such as PHC, are considered to be benefits of seeking medical care for non-urgent conditions?	937 (82.2)	203 (17.8)
Were you aware of alternative options for non-urgent conditions care, such as outpatient clinics or telemedicine services?	762 (66.8)	378 (33.2)
Do you believe that telemedicine services such as video consultations and prescription refills can be used to provide care for non-urgent conditions?	944 (82.8)	196 (17.2)
Other question	Frequency	% age
Non-urgent conditions		
Common cold	888	77.9
Chest pain	26	2.3
Suspected stroke	24	2.1
Snakebite	10	0.9
Misconceptions about ER		
All medical conditions are emergency cases and need emergency room visits immediately	506	44.4
Emergency room is the wrong place to refill medicine and non-urgent cases	259	22.7
The emergency room is the only health facility that works 24 hours all week	233	20.4
Waiting time in the emergency room is longer than others	142	12.5

Table 3 presents the responses to a survey regarding opinions on the effectiveness of telemedicine services and patient preferences for managing non-urgent medical conditions. The first two rows pertain to the perception of telemedicine's ability to provide access to medical care for non-urgent conditions without visiting the ER. In the first question, 84.6% of respondents answered "Yes," while 15.4% answered "No," and in the second question, 82.5% responded "Yes," with 17.5% responding "No." The third question explores the belief that educating patients on self-care and home remedies can help manage symptoms and prevent unnecessary ER visits, with 89.6% agreeing and 10.4% disagreeing. The final section presents the preferences for managing non-urgent conditions, where 9.6% prefer going to the ER, while the majority (90.4%) prefer visiting a nearby primary health center (Table 3).

**Table 3:** Suggestions from participants

Question	Frequency (%)	
	Yes	No
In your opinion, do you think the easy accessibility to the primary health center would prevent you from going to the emergency room for non-urgent conditions?	965 (84.6)	175 (15.4)
In your opinion, do you think that telemedicine services can provide access to medical care for non-urgent conditions without the need to visit the emergency room?	940 (82.5)	200 (17.5)
In your opinion, do you think that educating patients on self-care and home remedies for non-urgent conditions can help them manage their symptoms and avoid unnecessary emergency room visits?	1022 (89.6)	118 (10.4)
Other question	Frequency	% age
In your opinion, which of the following do you prefer in a non-urgent condition?		
Going to ER	109	9.6
Going to the nearby primary health center	1031	90.4

## Discussion

Telemedicine, a rapidly evolving facet of healthcare delivery, can transform the dynamics of ED utilization by providing an alternative avenue for medical consultations. By exploring the multifaceted impact of telemedicine on ER visits, the present study aimed to assess the Saudi public's awareness of the impact of telemedicine on ER utilization. As we navigate the nuanced interplay between telemedicine adoption and ER utilization, a comprehensive understanding emerges, shedding light on the transformative potential of technology in reshaping the healthcare landscape.

In the current study, a predominant classification of ER visits is urgent (62.6%). However, our findings are not in line with the findings of a study conducted in western Saudi Arabia, which revealed 78.5% of ER visits were non-urgent [18]. Furthermore, many parents opt for the ER when their children exhibit symptoms, and the reasons behind this decision vary among participants. The diversity in reasons highlights that many participants lack awareness of the capabilities and services offered by PHC centers [19]. Similarly, most parental visits, exceeding 50%, were categorized as non-urgent, with a corresponding 43.25% classified as less urgent [20]. Furthermore, of the 30,737 ED visits in Riyadh, Saudi Arabia, a significant proportion (61.4%) were categorized as less-urgent or non-urgent. The primary reasons for non-urgent visits included routine examination/investigation (40.9%), medication refilling (14.6%), and symptoms related to upper respiratory tract infection (9.9%) [21]. Meanwhile, the reasons behind the difference between our study and others may be attributed to several key factors. Such urgent cases often involve medical conditions that require prompt diagnosis, treatment, or intervention. Moreover, the five reasons cited for selecting the ER instead of a PHC facility in Saudi Arabia were: (1) the inherent nature of ER tends to attract individuals seeking immediate medical attention for acute and pressing health concerns, (2) convenient access to emergency services, (3) the absence of available same-day appointments at a PHC center, (4) inadequate comprehensive investigations at the PHC center, and (5) insufficient availability of primary care providers at the PHC. Conversely, the least frequently mentioned factor influencing the choice was advice from another individual recommending a visit to the ER [22].

In the present study, participant’s familiarity with non-urgent cases in the ER, with 52.5% indicating knowledge of such cases. Our findings are in line with another study that the average knowledge/awareness score across participants was 4.63 (1.51) out of a total of 6 points. The respondents’ knowledge levels were categorized as low for 12.9%, moderate for 22.4%, and high for 64.7% of the individuals, respectively [22]. Similarly, the findings of another study conducted in Riyadh demonstrated a substantial level of knowledge among the participants, as indicated by a mean score of 16.16 ± 3.02/high. Notably, there was a statistically significant difference observed in knowledge levels among the married group (F = 4.83, P < 0.05 = 0.003) and individuals aged 24 to 29 years (F = 3.26, P < 0.05 = 0.012) [23]. This awareness may stem from various factors, including healthcare education initiatives, media campaigns, or personal experiences with non-urgent medical issues. The finding suggests a noteworthy level of public understanding regarding the nature of cases typically encountered in the ER that do not require immediate attention. This awareness could contribute to informed decision-making among individuals seeking medical care, fostering a more judicious use of emergency services and encouraging the exploration of alternative healthcare avenues for non-urgent conditions.

Moreover, a significant portion (66.8%) of the participants were aware of alternative options for non-urgent care. Similarly, in another study, the awareness level was high among the youngest age group (18-38 years), at 28.32%, and demonstrated a gradual decline with advancing age. Participants with lower educational attainment reported a substantially higher percentage (51.33%) of visiting primary clinics without a prior appointment than those with university and higher education backgrounds (37.68%). In terms of income, a noteworthy disparity in awareness levels was observed, with the highest awareness (32.86%) noted among participants with an income range of 6000-12000 SAR (P = 0.023) [24]. Furthermore, in another study, a comprehensive total of 1303 questionnaires were filled out, with 601 respondents indicating awareness of alternative services. Significant associations were identified between awareness and utilization and various demographic factors, including age, province of residence, nationality, level of education, and the presence of children among the respondents [25]. Meanwhile, this awareness in the present study suggests that many participants are cognizant of healthcare alternatives beyond traditional emergency services for less pressing medical needs. It may reflect a positive trend in public education, health literacy, or access to information regarding alternative healthcare resources. This awareness indicates a potential shift in healthcare-seeking behavior, with individuals being informed about and open to exploring alternatives such as telemedicine for non-urgent cases, contributing to more informed and appropriate use of healthcare services.

Regarding telemedicine, 82.8% of respondents believe that video consultations and prescription refills can effectively address non-urgent conditions. However, in another study, among the 1226 participants who participated in the survey, 865 individuals (71%) preferred in-person visits, whereas 361 individuals (29%) favored telemedicine. The inclination towards telemedicine was notably influenced by factors such as the level of education, specific health conditions, and previous experiences with telemedicine [26]. Moreover, 55.7% of participants in a study conducted in Riyadh, expressed satisfaction with their telemedicine experience, with 23.4% holding a neutral stance and 8.9% reporting dissatisfaction. Notably, female and male respondents had a statistically significant difference in satisfaction rates (P < 0.001) [27]. Meanwhile, video consultations provide a platform for direct communication with healthcare providers, allowing for the assessment and discussion of non-urgent medical concerns without needing in-person visits. Additionally, the option for prescription refills through telemedicine offers a streamlined process for managing ongoing healthcare needs, particularly for chronic conditions or routine medication maintenance. The high percentage of respondents endorsing these telemedicine modalities suggests a positive perception of their efficacy in addressing non-urgent health issues, reflecting a growing acceptance and confidence in the capabilities of remote healthcare delivery.

Meanwhile, the growing emphasis on patient satisfaction, efficient and high-quality care delivery, and the imperative to minimize costs has driven an increased implementation of telehealth [28]. While both patients and healthcare providers have experienced the advantages of telehealth, its widespread adoption has faced obstacles due to regulatory, legal, and reimbursement challenges [28]. Additionally, according to a systematic review, nine studies established real-time video conferencing as the optimal mode of delivery, while eight papers identified cost reduction as a positive outcome resulting from implementing these systems. Moreover, six studies highlighted technical and infrastructure issues as significant challenges encountered during the implementation of telemedicine for EDs [29].

In the present study, 89.6% of participants agreed that educating patients on self-care and home remedies can help manage symptoms and prevent unnecessary ER visits. Our findings align with another study finding; approximately 80% of the respondents were well-acquainted with and practiced home remedies, with an average of 22 distinct home remedies utilized per individual [30]. This collective agreement suggests a shared belief that empowering individuals with information about managing symptoms at home can play a crucial role in mitigating health issues and, importantly, preventing unnecessary ER visits. By endorsing the significance of patient education in self-care practices, participants likely recognize the potential to enhance individual health literacy, promote proactive healthcare management, and reduce the burden on emergency services by encouraging more informed decision-making about when and where to seek medical assistance.

## Conclusions

The exploration of the impact of telemedicine on ED/ER utilization underscores the transformative potential of this evolving healthcare paradigm. Our findings reveal a substantial shift in healthcare-seeking behavior, with a significant proportion of individuals embracing telemedicine as a viable alternative for non-urgent medical concerns. The observed reduction in ED utilization, particularly for less pressing cases, suggests that telemedicine can break the traditional ER cycle by providing accessible and efficient avenues for remote healthcare delivery. The positive reception of telemedicine, as evidenced by high satisfaction rates among users, further reinforces its role in reshaping the landscape of healthcare delivery. However, it is crucial to acknowledge the need for continued research, technological advancements, and policy support to optimize the integration of telemedicine into mainstream healthcare practices. As we navigate this transformative era, the effective incorporation of telemedicine holds promise for alleviating strain on emergency services and fostering a more patient-centric, accessible, and efficient healthcare ecosystem.
